# QuickStats

**Published:** 2014-09-26

**Authors:** FROM THE NATIONAL CENTER FOR HEALTH STATISTICS

**Figure:**
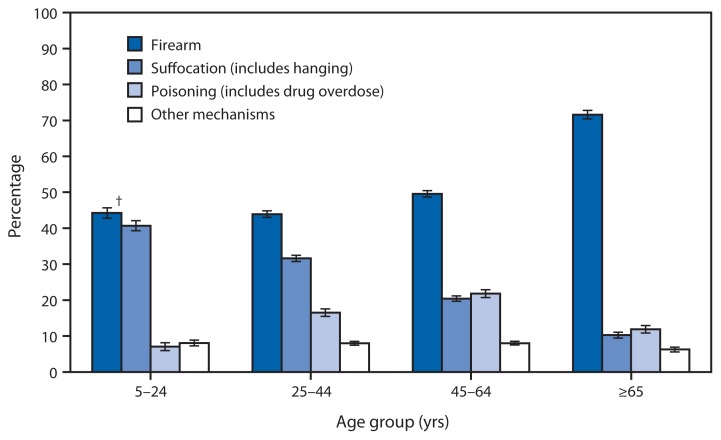
Percentage of Suicide Deaths, by Mechanism* and Age Group — United States, 2011 * Suicide deaths were categorized by mechanism of injury using the following *International Classification of Diseases, Tenth Revision* codes: firearm (X72–X74), suffocation (X70), poisoning (X60–X69) and other mechanisms (U03, X71, X75–X84, and Y87.0). ^†^ 95% confidence interval.

In 2011, firearm was the leading mechanism for suicide deaths for all age groups, ranging from 44% of suicides among persons aged 5–24 years to 72% of suicides among persons aged ≥65 years. Suffocation was the second leading mechanism in the two younger age groups (41% of suicides among persons aged 5–24 years and 32% of suicides among persons aged 25–44 years). In contrast, poisoning was the second leading mechanism (22%) among adults aged 45–64 years and those aged ≥65 years (8%).

**Source:** National Vital Statistics System mortality data. Available at http://www.cdc.gov/nchs/deaths.htm.

**Reported by:** Yahtyng Sheu, PhD, ydq6@cdc.gov, 301-458-4354; Li-Hui Chen, PhD; Holly Hedegaard, MD.

